# Beyond Immunotherapy: Seizing the Momentum of Oncolytic Viruses in the Ideal Platform of Skin Cancers

**DOI:** 10.3390/cancers14122873

**Published:** 2022-06-10

**Authors:** Dimitrios C. Ziogas, Anastasios Martinos, Dioni-Pinelopi Petsiou, Amalia Anastasopoulou, Helen Gogas

**Affiliations:** First Department of Medicine, School of Medicine, National and Kapodistrian University of Athens, 115 27 Athens, Greece; ziogasdc@gmail.com (D.C.Z.); anastasis.martinos2@gmail.com (A.M.); dionipetsiou@gmail.com (D.-P.P.); amanastasop@yahoo.gr (A.A.)

**Keywords:** oncolytic viruses, immunotherapy, skin cancer, melanoma, talimogene laherparepvec (T-VEC)

## Abstract

**Simple Summary:**

Oncolytic viruses (OVs) are the most innovative and promising class of intratumoral immunotherapies. The broad immunogenic landscape of skin cancer, accessible to intralesional infusion and available for direct response assessment, seems to be an ideal platform to expand the role of OVs. The established efficacy of immune checkpoint inhibitors (ICIs) in this field and their hypothetical synergy with OVs have generated expectations for their combined use beyond the current immunotherapy achievements. Despite the recent negative phase III results of the MASTERKEY-265 trial for the combination of T-VEC plus pembrolizumab, such projects, including different ICIs and various natural or genetically modified OVs, continue to attract considerable interest, with numerous clinical trials underway for all the subtypes of skin cancer. To date, the majority of studies confirm the safety of tested OVs in patients with advanced skin cancers but cannot clearly prove whether these viral agents add any therapeutic benefit in the standard ICI-based approach. The aim of this overview is to present the main findings related to the examined OV-containing regimens at pre-clinical and clinical levels, and to discuss the previous failures as well as the future perspectives of oncolytic virotherapy.

**Abstract:**

Despite the durable remissions induced by ICIs and targeted therapies in advanced melanoma and non-melanoma skin cancers, both subtypes usually relapse. Many systematic therapies have been tested to increase efficacy and delay relapse in ICIs, but their success has been limited. Due the feasibility of this approach, skin cancers have become the ideal platform for intralesional infusions of many novel agents, including oncolytic viruses (OVs). Talimogene laherparepvec (T-VEC) was the first FDA-approved OV for the treatment of unresectable melanoma and this virus opened up further potential for the use of this class of agents, especially in combination with ICIs, in order to achieve deeper and longer immune-mediated responses. However, the recently announced phase III MASTERKEY-265 trial was not able to confirm that the addition of T-VEC to pembrolizumab treatment improves progression-free or overall survival over the use of pembrolizumab alone. Despite these results, numerous studies are currently active, evaluating T-VEC and several other OVs as monotherapies or in regimens with ICIs in different subtypes of skin cancer. This overview provides a comprehensive update on the evolution status of all available OVs in melanoma and non-melanoma skin cancers and summarizes the more interesting preclinical findings, the latest clinical evidence, and the future insights in relation to the expected selective incorporation of some of these OVs into oncological practice.

## 1. Introduction

Skin cancer, including melanoma and non-melanoma subtypes (NMSC), represents the most common malignancy among Caucasians in the Western world [1,2,3]. Although in the early stages both subtypes remain curable after surgical resection and adjuvant therapy, where appropriate, in metastatic settings these tumors eventually relapse and retain a poor prognosis [4,5]. Both melanoma and NMSCs, mainly including cutaneous squamous cell carcinoma (CSCC) and basal cell carcinoma (BCC), have been highlighted for their high tumor mutational burden (TMB) and their abundant tumor neoantigens [6]. The deeper comparison of melanoma with NMSCs has progressively revealed many proteomic, genomic, and immunological differences associated with cancer development and their recognition has subsequently helped improve the understanding of the dynamic interplay in the tumor microenvironment (TME) between malignant cells and those regulating either innate or adaptive immunity [7]. Knowledge on this immune background provided the basis for the introduction of immune checkpoint inhibitors (ICIs), which have drastically changed the way in which skin cancers are treated [5,8]. In metastatic melanoma, the combination of nivolumab plus ipilimumab, according to an extended follow-up of 6.5 years, offers the longest median OS among phase III trials (72.1 months) [9]. In advanced CSCC, pembrolizumab (since June 2020) [10] and cemiplimab (since September 2018) [11] have been proven preferred first-line options over conventional chemotherapy, and in unresectable BCC, cemiplimab received FDA approval (in February 2021) to overcome resistance to hedgehog inhibitors [8]. However, more information regarding the neoantigen burden, the features of immune cell infiltrates, and the duration of immune-mediated responses are required in order to optimize the role of immunotherapy in melanoma and NMSCs. New immunotherapeutic agents with non-overlapping toxicity profiles, such as complementary ICIs and oncolytic viruses (OVs), are currently under investigation to overcome the present limitations of ICIs (improving the T-cell exhaustion, prolonging the duration of response, and delaying resistance) and to tailor the treatment in response to the continuous phenotypic and antigenic modifications that characterize skin cancer cells. For instance, the co-inhibition of LAG-3 and PD-1 with the combination of relatlimab and nivolumab recently provided a greater benefit than single anti-PD-1 inhibition in untreated patients with metastatic or unresectable melanoma without new safety signals [12].

In this context, the use of viruses is not new. In more than 100 years of development, clinical experience on OVs has recorded periods of highs (1950s and 1960s) and lows (1970s and 1980s) due to the poor results and severe toxicities observed in the initial human trials [13,14]. The advances in molecular biology and genetic editing in the late 20th century allowed scientists to isolate viruses with a natural selectivity for tumor cells and to genetically modify some of them in order to generate tumor-specific replication and reduce virulence to normal cells. A major milestone in OV development occcurred in 2015, when the *Food and Drug Administration* (FDA) approved talimogene laherparepvec (T-VEC) as the first engineered oncolytic herpes simplex virus type 1 (HSV-1) for the treatment of recurrent melanoma after initial surgery [15,16]. Mechanistically, OVs, in their natural or “armed” status, can selectively infect cancer cells and lyse them, releasing tumor-derived antigens (TDAs), viral pathogen-associated molecular pattern signals, cellular danger-associated molecular pattern signals, and cytokines [17,18]. Using tumors as a source of antigens, intralesional OV injection initiates the local recruitment of dendritic cells (DCs) into the tumor microenvironment (ΤΜΕ), accelerates cross-presentation, and subsequently primes T cells for a systemic polyclonal antitumor response, potentially addressing intra- and intertumoral heterogeneity [19,20]. The OV-induced upregulation of IFN-a and the deletion/inhibition of certain viral genes known to downregulate MHC class I expression (for example, the deletion of the *ICP47* gene of HSV-1) can further promote antigen presentation processing [16,21]. OVs can be “armed” with transgenes expressing immunostimulatory cytokines and chemokines (e.g., T-VEC expresses GM-CSF) or can release some other costimulatory ligands that support tumor-specific T cell expansion and activation inside and outside the tumor site [18]. Thus, the locally induced infection leads to tumor lysis and shrinkage but, in parallel, triggers a more systemic immune reaction far away from the injected lesions [15,16,18].

These multiple effects of OV vaccination are greatly dependent on the host immune response, and this response may significantly vary among individual patients, different timepoints of treatment, different types of malignancies, and different types of administered viruses. There is still a lot to be learned about the individualized immunological response to OV therapy. The broad immunogenic landscape of skin cancer, accessible to intralesional infusion and available for the direct assessment of the response, seems to be an ideal platform to further examine the role of OVs. Moreover, the established efficacy of immunotherapy in this field and the relatively safe profile of oncolytic virotherapy have generated expectations for the use of their combinations beyond the current achievements. However, the recent results of the MASTERKEY-265/KEYNOTE-034 study [22] moderated the initial enthusiasm after T-VEC approval and raised some major concerns regarding the exact mechanism of action of OVs and their future potential. Currently, numerous clinical trials are active, evaluating the use of T-VEC and several other OVs as monotherapies or in combinations with ICIs, in order to allocate these viral agents in the treatment algorithms of skin cancers. Here, we provide an update on the evolution status of all OVs under clinical testing and summarize the preclinical findings, the current evidence, and the future perspectives for each one of them. In parallel, we further focus on those OV-regimens with the most promising results, and discuss the limitations that hinder their broader oncological implication.

## 2. The Evolution of OVs: From the Preclinical Background of HSV Selection to the Current Clinical Status of Tested OVs

Even before the first OV approval, herpesviruses were the most commonly investigated candidates for oncolytic virotherapy in skin cancers (e.g., T-VEC, RP-1, and HF-10), with the vast majority of preclinical and clinical data gathered in this area. Table 1 summarizes the main favorable characteristics of these herpesviruses. Other investigational viruses include adenoviruses (such as TILT-123, ICOVIR-5, and ONCOS-102) [23,24,25], rhinoviruses (such as PSVRIPO) [26], and coxsackieviruses (such as CAVATAK) [27,28], as well as ECHO viruses [29,30].

The rationale behind the selection of HSV as the prominent viral vector for producing an optimal “armed” OV is based on some main viral characteristics, such as selective viral replication, low toxicity and virulence, as well as the availability of effective anti-herpetic medications in case of an undesired spread. Since the publication of the complete genomic DNA sequence of HSV-1 in 1988 [31], a number of studies have focused on the complete open-reading frames and the identification of individual HSV genes in viral pathogenicity [32,33]. The deep knowledge of HSV gene products involved in the interaction with the host cell and evasion from the immune system has allowed the development of therapeutic HSV-derived vectors for several human diseases. In contrast to other viruses that bind to target cells via a single receptor, HSV has four entry receptors that allow it to infect and replicate into almost all cancer cell lines, with potency against many different types of tumors, even in non-replicating neuronal cells. This multi-receptor property helps HSV to infect and kill infected cells more rapidly, compared to adenoviruses, making the development of resistance more difficult [15,34]. However, other modalities may be also implicated, since Wang et al. found that the entry of HSV in neuroblastoma cells is independent of the sum of all four known major HSV receptors, suggesting that the cellular anti-viral response, not virus entry, is the key determinant of sensitivity to virotherapy [35].

The tumor-selective replication of HSV is built on the deletion of two genes, ICP34.5 and ICP47. In a specific clinical isolate JS1 strain, HSV-1 with a deleted ICP34.5 gene showed increased cell lysis in all human tumor cell lines tested, compared to a laboratory strain [16]. The deletion of the ICP34.5 gene enables viral replication and direct tumor-specific cell lysis in several tumor models by attenuating in parallel the natural neurovirulence. This tumor-specific cell killing capacity of ICP34.5- HSV has been further enhanced by the co-deletion of the ICP47 gene [16]. As a DNA virus, HSV also retains the potential for incorporating foreign DNA, and can work as an identical vector for being “armed” with transgene modifications. For instance, the gene encoding human or mouse GM-CSF was experimentally inserted into the JS1/ICP34.5-/ICP47-HSV vector and its expression was found to trigger host immunity by promoting the differentiation of progenitor cells into DCs [16]. Thus, the local infection induced by the intralesional infusion of such an “armed” OV could cause a more systemic GM-CSF-mediated response [36]. These constructed ICP34.5-/ICP47-/GM-CSF+ herpesviruses were tested in vitro in human tumor cell lines and in vivo in mice, demonstrating antitumor effects significantly better than those of viruses not containing any of the described modifications. The application of this strategy led to the prototypical viral agent in this class, JS1/ICP34.5-/ICP47-/GM-CSF+ HSV-1, or more commonly, T-VEC. Starting from T-VEC, we present below the main preclinical and clinical findings for each of the available viral agents under testing in skin cancers (Table 2 and Table 3).

### 2.1. T-VEC (OncoVEXGM-CSF)

**Figure 1:**
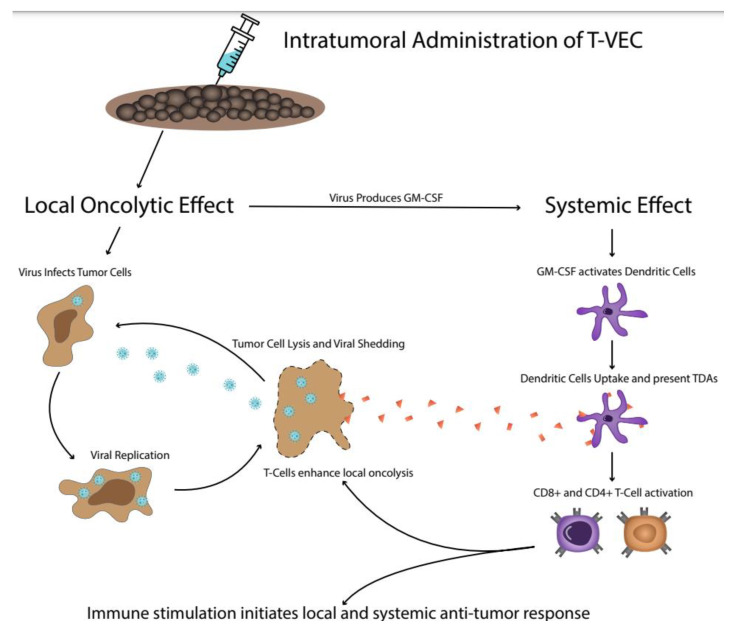
The dual anti-tumor mechanism of action of T-VEC. Initially, T-VEC infects tumor cells and replicates intracellularly, causing cell lysis and the release of TDAs. In addition to the local inflammation, the genetically-induced production of GM-CSF attracts and activates dendritic cells, which uptake the TDAs and activate CD4+ and CD8+ T cells, initiating a systemic anti-tumor response. T-VEC = Talimogene laherparepvec; TDAs = tumor-derived antigens; GM-CSF = granulocyte-macrophage colony-stimulating factor.

T-VEC was the first approved genetically engineered OV derived from a clinical HSV-1 strain (JS1) with the deletion of the ICP34.5 and ICP47 genes and the addition of GM-CSF expression. According to preclinical data, it can enter cancer cells by using any of the four surface-bound nectin 1 HSV receptors and can preferentially replicate inside tumor cells by exploiting oncogenic and viral signaling pathways [37,38]. The lysis of infected cells is followed by the release of TDAs, antigen presentation via MHC-1 molecules, and subsequent T-cell priming and activation. Thus, the intralesional injection of T-VEC can reverse the immunologically “cold” TME and the concurrent expression of GM-CSF can further trigger a systemic immune-mediated antitumor response [16,19] (Figure 1).

Preclinical models in A20 lymphoma mice showed that T-VEC can display high direct efficacy in the injected tumors (with a 70–100% regression rate), and additionally an abscopal effect (with regression in 50–60% of non-injected lesions) [16,39,40]. More specifically, Cooke et al. noted that treatment with T-VEC caused tumor regression and complete cures in 10/10 injected tumors and in 5/10 contralateral tumors when dosed at 3 × 10^6^ pfu/mouse [39]. Interestingly, viral DNA was detected in all injected tumors, but in only 1/16 contralateral tumors [39]. Although in mice, these data supported the notion that direct oncolysis was not the mechanism responsible for the regression of distant non-injected tumors. After flow cytometry analysis of T-cell populations from mice treated with T-VEC, cell surface activation markers were detected to be expressed at higher levels compared to those prior to intralesional administration and compared to vehicle controls [40]. Moreover, the T-cell-mediated response generated by the viral vaccination was found to protect animals against the re-injection with tumor cell lines, revealing an overall activation of immunological memory [40]. Using a murine version of T-VEC, Moesta et al. described the local and systemic antitumor efficacy of T-VEC alone or in combination with an ICI in syngeneic contralateral murine tumor models (A20 lymphoma and CT-26 colon carcinoma mice) [41]. The T-VEC/pembrolizumab combination displayed 80% complete regressions in injected tumors and 20% complete regressions in distant lesions [41]. The analysis of peripheral blood confirmed that the combination increased the percentage of activated CD8+ T cells. Similarly, the combination of T-VEC with an anti-CTLA-4 ICI resulted in increased tumor-specific CD8^+^ T cells and systemic efficacy in A20 and CT-26 contralateral murine tumor models [41]. Although the majority of injected tumors in T-VEC treated animals showed complete regression at all doses, contralateral tumors showed no response at the lower dose, whereas the medium and high doses showed complete responses in half tumors and growth delays in the other half [41].

In clinical setting, T-VEC has been studied either as a single agent or in combinatorial regimens, mainly with ICIs, in advanced melanoma or NMSCs (Table 2). In the first-in-human phase I study, the safety and the dose schedule of T-VEC was evaluated in 30 pretreated patients with cutaneous or subcutaneous deposits from various tumors, including breast (*n* = 14), head and neck (*n* = 5), and colorectal cancers (*n* = 2) and malignant melanoma (*n* = 9) [42]. Using a multi-dosing protocol (an initial dose of 10^6^ pfu/mL, followed by multiple higher doses up to 10^8^ pfu/mL), T-VEC was proven to be safe, with hyperpyrexia, local inflammation, and erythema being the main side effects [42]. The duration of local reactions suggested that dosing every 2 to 3 weeks was acceptable. Post-treatment biopsies showed that 19 of 26 patients contained residual tumors, of whom 14 showed tumor necrosis, which in some cases was extensive, or apoptosis. In all cases, areas of necrosis were strongly stained for HSV. The overall responses were three patients with stable disease (SD), six patients with tumors flattened (injected and/or uninjected lesions), and four patients with inflammation in their uninjected lesions. The injected tumors became inflamed in nearly all cases. The ongoing phase I clinical trial (NCT03064763) in Japanese subjects with stage IIIB-IV melanoma reported four serious AEs in two of six DLT-evaluable patients: infectious enteritis, worsening of benign prostatic hyperplasia, epiglottitis, and pneumonia, and additional data from other 12 enrolled patients are being awaited [43].

In the next level, a phase II single-arm trial evaluated the efficacy and safety of T-VEC in 50 patients with unresectable, stage III_C_/IV melanoma (NCT00289016) [44]. Treatment involved the intratumoral injection of up to 4 m of 10^6^ pfu/mL of T-VEC (reported in the trial as JS1/ICP34.5-/ICP47-/GM-CSF+), followed 3 weeks later by up to 4 mL of 10^8^ pfu/mL every 2 weeks for up to 24 treatments. Of the 50 patients included in the core study, 74% had received prior nonsurgical treatment for active disease, including dacarbazine/temozolomide or IL-2 [44]. Patients received a median of six T-VEC injections and Aes were limited mostly to transient flu-like symptoms. According to the Response Evaluation Criteria in Solid Tumors (RECIST), the ORR was 26% (CR = 16% and PR = 10%), including regressions of both injected and distant visceral lesions [44]. Ninety-two percent of responses had been maintained for 7 to 31 months. Twenty percent of patients had SD for more than 3 months, and two additional patients had surgical CR. The OS was 58% at 1 year and 52% at 2 years. At 24 and 72 h after the first injection, 102 swabs were taken from injection sites in 19 patients and only one swab tested positive for the virus (at a low level), suggesting that viral shedding was rare in this setting. None of the 78 urine samples collected at 1–48 h in 13 patients tested positive for viral DNA via polymerase chain reaction analysis. Kaufman et al. described the immunophenotypic analysis of T cells derived from tumor samples and from peripheral blood in a subset of patients treated in this phase II clinical trial [45]. Treatment with T-VEC was found to decrease the levels of suppressor cell populations, including CD4+Tregs, CD8+T suppressor cells, and MDSCs, in injected lesions compared with non-injected lesions in the same (and different) melanoma patients, whereas in parallel this treatment increased the levels of local and systemic MART-1-specific CD8+effector cells in tumors undergoing regression compared with melanoma patients not treated with OV. Another phase II, multicenter, open-label study in patients with unresectable stage III_B_/IV melanoma, evaluated whether baseline or changes in intratumoral CD8^+^ T-cell density were correlated with the T-VEC clinical response [46]. After a median follow-up of 108 weeks, ORR and CRR were 28% and 14%, respectively, in the overall population and 32% and 18% in patients with stage III_B_/IV_M1a_ disease. Serious Aes were observed in 29.73% of the subjects. Exploratory analyses showed a 2.4-fold median increase in CD8^+^ T-cell density in non-injected lesions from baseline to week 6, particularly in those subsets expressing granzyme B and checkpoint markers (PD-1, PD-L1, CTLA-4), together with an increase in helper T cells. Consistent with T cell infiltration, an increase in the adaptive resistance marker PD-L1 in non-injected lesions was observed. Neither baseline nor other changes in CD8^+^ T-cell density were correlated with ORR, changes in tumor burden, DoR, or the durable response rate [46]. The virulence of T-VEC was examined in 61 treated patients with stage IIIc/IV melanoma with ≥1 injection and afterwards they were tested for possible viral DNA detection (NCT02014441) [47]. T-VEC DNA was detected only on 8 of 740 swabs (1.1%) from the surface of injected lesions. Only three close contacts reported signs and symptoms of suspected herpetic origin but without detectable viral DNA. The study concluded that T-VEC is unlikely to be transmitted with the appropriate use of occlusive dressings [47].

The positive results of phase I and II studies supported the initiation of the randomized open-label phase III OPTiM trial (NCT00769704, EudraCT 2008-006140-20). Recently, the final planned analysis of OPTiM, including 437 patients with unresectable stage IIIB–IVM1c melanoma [48] showed that T-VEC was associated with durable CRs and prolonged survival compared to GM-CSF as the control arm, and remained well tolerated. T-VEC was administered at a concentration of 10^8^ pfu/mL, injected into one or more skin or subcutaneous tumors on days 1 and 15 of each 28-day cycle for up to 12 months, whereas GM-CSF was administered at a dose of 125 μg/m2/day subcutaneously for 14 consecutive days, followed by 14 days of rest, in 28-day treatment cycles, for up to 12 months. At a median follow-up of 49 months, the median OS was 23.3 and 18.9 months in the T-VEC and GM-CSF arms, respectively (unstratified HR = 0.79; 95%CI: 0.62–1.00; *p* = 0.0494). ORR was 31.5% (CR = 16.9%) and 6.4% (CR = 0.7%) and DRR was 19.0% and 1.4% (unadjusted OR = 16.6; 95%CI: 4.0–69.2; *p* < 0.0001), respectively. Among patients with a CR, 88.5% were estimated to survive at a 5-year landmark analysis. The efficacy of T-VEC was more pronounced in stage III_B_/IVM1a melanoma. The safety reporting was consistent with the primary OPTiM analysis. The most common serious TRAEs noted were gastrointestinal hemorrhage, pyrexia, cellulitis, and tumor pain. Of the patients receiving T-VEC, 96.58% manifested non-serious Aes, whereas the respective percentage for those receiving GM-CSF was 88.19%. The biomarker analysis in the OPTIM study showed that treatment with T-VEC can cause an increase in the levels of effector CD8^+^ T-cells and NK cells but not in those of macrophages. Baseline tumor CD8^+^ density was found to be correlated with DR but not overall response. These data further supported the considerations related to combining T-VEC with ICIs [49]. In total, this phase III trial confirmed that T-VEC had both a direct oncolytic effect in injected tumors and a secondary immune-mediated anti-tumor effect in uninjected lesions, leading in 2015 to the FDA approval of T-VEC as the first viral agent for the treatment of advanced melanoma.

Regarding the combination of T-VEC with ICIs, Chesney et al. presented the extended follow-up of a phase II, open-label multicenter trial (NCT01740297) on the combination of T-VEC and ipilimumab versus ipilimumab alone in 198 patients with unresected, stage III_B_/IV melanoma [50]. The T-VEC plus ipilimumab combination continued to provide durable and statistically superior ORR versus ipilimumab alone (36.7% vs. 16.0%; *p* = 0.002). Interestingly, responses were not limited to injected lesions; visceral lesion decreases were observed in 52% of patients in the combination arm and 23% of patients in the ipilimumab arm. mPFS was numerically longer with the combination compared to ipilimumab alone (13.5 vs. 4.5 months; *p* = 0.159), whereas mOS was not reached in either arm (*p* = 0.480). OS may be confounded by subsequent anticancer therapies, as 45.9% in the combination arm and 64% in the ipilimumab arm received subsequent anticancer therapy, with the median time from randomization to the first subsequent therapy being 27.7 and 8.3 months, respectively. The most frequently occurring Aes included fatigue (combination, 59%; ipilimumab alone, 42%), chills (combination, 53%; ipilimumab alone, 3%), and diarrhea (combination, 42%; ipilimumab alone, 35%). The incidence of Aes of grade ≥ 3 was 45% and 35%, respectively. Three patients in the combination arm had fatal Aes but none were treatment-related. These data indicate that the OV/ICI combination showed greater antitumor efficacy without additional safety concerns versus ICI alone.

Two phase II studies have recently presented their rationales and their designs for the combination of T-VEC with pembrolizumab in patients with metastatic/unresectable melanoma after initial anti-PD-1 failure. First, the phase II, open-label, single-arm, multicenter MASTERKEY-115 trial (NCT04068181) has enrolled approximately 100 patients in four cohorts (Cohort 1: rechallenge with anti-PD-1 in a locally recurrent or metastatic setting after experienced PD within 12 weeks of the last anti-PD-1 dose with best prior response = SD; Cohort 2: similar to cohort 1 with best prior response = CR or PR prior to confirmed PD; Cohort 3: adjuvant anti-PD-1 and disease-free for <6 months; Cohort 4 adjuvant anti-PD-1 and disease-free for ≥6 months prior to relapse). Eligible subjects should have histologically proven unresectable or metastatic stage III_B_-IV_M1_ melanoma, measurable and injectable disease, ECOG PS 0-1, and prior anti-PD-1 (≥2–3 consecutive cycles, immediate prior treatment before enrollment). The results of MASTERKEY-115 trial will be presented in the ASCO 2022 meeting. The second phase II study examines the use of T-VEC plus pembrolizumab in patients with advanced melanoma whose disease progressed after prior therapy with a PD-1/PD-L1 ICI. In contrast to the eligibility criteria of the other study, in these patients the immediately previous ICI-based therapy (within 56 days prior to registration) must offer no confirmed PR or CR. Subjects in cohort A must have at least one measurable visceral lesion, and in cohort B subjects must not have visceral lesions. A total of 36 subjects will be enrolled in cohort A and 25 subjects in cohort B, with a Simon 2 stage design [51].

This combination of T-VEC and pembrolizumab has entered into focus even for treatment-naïve patients with unresectable or metastatic melanoma. In the long-term follow-up of the phase Ib part of the MASTERKEY-265 study (median = 58.6 months), mDoR, mPFS, and mOS were not reached for patients treated with the pembrolizumab/T-VEC combination, and 4-year PFS% and OS% have held stable since the three-year analysis (55.9% and 71.4%, respectively). The CR rate remained 43% (9/21 patients). Of patients who achieved a CR or PR, 92% had better OS (*p* = 0.0056) compared to those who did not respond [52]. No additional safety signals were detected. However, the largest study on OVs to date, the phase III, randomized, double-blind MASTERKEY-265/KEYNOTE-034 trial (NCT02263508) was not able to prove the superiority of the combination of T-VEC and pembrolizumab versus pembrolizumab monotherapy in patients with stage III_B_/IVM_1c_ melanoma [22]. T-VEC was administered intratumorally at a dose of ≤4 × 10^6^ pfu, followed by ≤4 × 10^8^ pfu 3 weeks later and every 2 weeks until dose 5, and every 3 weeks thereafter. Pembrolizumab was administered intravenously at the standard dose of 200 mg every 3 weeks. The results of primary PFS and interim OS analyses in the MASTERKEY-265/KEYNOTE-034 study were reported during the ESMO 2021 meeting. A total of 692 previously untreated melanoma patients were randomized (T-VEC/pembrolizumab: 346, placebo/pembrolizumab: 346); 6.9% had stage IVM1c disease, 32.7% had high LDH levels, and 64.9% had PD-L1+ status. After a median follow-up of 31.0 months, mPFS was estimated to be 14.3 months for the T-VEC/pembrolizumab arm and 8.5 months for the placebo/pembrolizumab arm (HR = 0.86, 95%CI: 0.71–1.04, *p* = 0.13) [22]. ORR was 48.6% (CRR = 17.9%) and 41.3% (CRR = 11.6%) for the two treatment arms, respectively. mOS was not reached for the OV/ICI combination and was 49.2 months for ICI alone (HR = 0.96, 95%CI: 0.76–1.24, *p* = 0.74). In the primary analysis, OS was not expected to achieve statistical significance. DRR was 42.2% in the T-VEC/pembrolizumab arm and 34.1% for the placebo/pembrolizumab arm. There was no difference in DoR between arms (HR = 1.04, 95%CI: 0.67–1.60, *p* = 0.87). The safety of the T-VEC/pembrolizumab combination was consistent with the known profiles of each agent, with more than grade 3 TRAEs in 21.2% and 16% of patients, respectively.

In the neoadjuvant setting, the first and largest ongoing trial of T-VEC (NCT02211131) recently announced the three-year results of the interim analysis of 150 patients with resectable stage III_B_/III_C_/IV_M1a_ melanoma that were randomized to receive six doses of T-VEC prior to surgery (*n* = 76) or to undergo to immediate surgical resection (*n* = 74) [53]. This study had already met its primary endpoint of a 25% reduction in the risk of disease recurrence at 2 years for neoadjuvant T-VEC plus surgery versus upfront surgery for patients with resectable stage IIIB-IVM1a melanoma [54]. After an extended follow-up, the three-year RFS% results were 46.5% vs. 31.0% (HR = 0.67, *p* = 0.043). This difference is clearer in the sensitivity analysis, removing the potential effect of subsequent anticancer therapy on RFS, giving three-year RFS% estimates of 49.1% vs. 22.9% (HR = 0.60, *p* = 0.022). The 3-year OS% results were 83.2% and 71.6% (HR = 0.54, *p* = 0.061) [53]. Increased CD8^+^ density was correlated with clinical outcomes in an exploratory analysis. Interestingly, the mortality rate was higher for the upfront-operated patients (20/74 vs.11/76). Patients treated with T-VEC experienced more commonly serious TRAEs (17.81%) compared to those of the surgical arm (2.90%). The most common grade ≥3 TRAEs among subjects treated with T-VEC prior surgery were cellulitis (2.74%), gastrointestinal disorders (2.74%), pyrexia/influenza (2.74%), and pain/post-operative wound infection (2.74%). No new safety signals were detected. The neoadjuvant strategy of T-VEC prior to surgical resection seems to be more effective than surgery alone, decreasing the possibility of disease recurrence by at least 25%. The final analysis will occur at 5 years. Finally, Tulokas et al. investigated the use of T-VEC in combination with isolated limb perfusion (ILP) (NCT03555032 NCT02094391 NCT03685890 NCT03555032) [55]. The trial enrolled 60 patients, who received ILP as a treatment for limb-limited melanoma. It consisted of two arms, the first one including the administration of ILP plus nivolumab and the second one including ILP plus a placebo. Specifically, one day before planned ILP administration, the patient would receive nivolumab or a placebo, respectively. Whenever toxicity was observed among the participants, it was generally transient. Notably, the median melanoma-specific-survival was higher in younger patients (<69 years) than in older patients (>69 years) [55].

As another version of T-VEC, a GS-CMF-expressing HSV-1-derived OV, OrienX010 was recently tested at four dose groups—10^6^ pfu, 10^7^ pfu, 10^8^ pfu, and 4 × 10^8^ pfu—in Chinese patients with no observed DLTs in all cohorts and sufficient efficacy outcomes (ORR = 28.6%, DCR = 57.1%, mPFS = 3.0 months, and mOS = 17.4 months) [56]. Intratumoral OrienX010 was also examined in combination with intravenous toripalimab 3 mg/kg every 2 weeks for 4–6 doses as a neoadjuvant therapy in patients with potentially resectable stage III_B_/IV_M1a_ acral melanoma (phase Ib, NCT04197882) [57]. Toripalimab is a selective, recombinant, humanized monoclonal antibody against PD-1, developed in China [58]. After surgical resection, toripalimab was continued as an adjuvant immunotherapy for up to 1 year. Of 24 patients who completed neoadjuvant treatment, 21 (88%) underwent surgery and 3 (12%) patients did not undergo surgery due to disease progression, whereas the other six enrolled patients were still receiving neoadjuvant treatment at data cut-off. Of 21 operated patients, 3 (14%) showed a pCR and 14 (67%) showed a pPR. Pathologic responses were associated with greater lymphoid infiltrate, hyaline fibrosis, and a decrease in Ki-67 expression in the metastasis. After a median follow-up of 8.9 months, none of the operated patients experienced a melanoma recurrence [57]. The OV/ICI treatment was well tolerated, with all patients having at least one TRAE and fever being the most common side effect. Three (10%) patients had a grade 3–4 TRAE, including one alanine aminotransferase increase and two wound infections [57]. In another phase Ib setting (NCT04206358), the same OV was intra-hepatically injected in patients with melanoma liver metastases, in combination with intravenous toripalimab, showing remarkable pathological responses in melanoma liver metastases [59]. Via investigator-assessment of the 15 eligible subjects, the response rate was 40% (6/15) for injected lesions, 28.5% (4/14) for non-injected lesions in the liver, and 23% (3/13) for extra-hepatic metastasis. At 8–12 weeks, injected lesions were biopsied: 30% showed no residual disease based on immunohistochemistry, 46.7% had an impressive TIL infiltration compared with the baseline absence of TIL infiltration, whereas a large number of plasma cells, histiocytes, and pigments were also found with hyaline fibrosis. mPFS was not reached and all Aes were grade ½: pyrexia 86.7%, rigor 66.7%, elevated transaminase 53.3%, nausea/vomiting 40.0%, and fatigue 26.7% [59].

Clinical trials including T-VEC have been also set up for other non-melanoma cancers with squamous origins [60,61] (Table 3). Of these trials, only a phase Ib/III study (NCT02626000) has published the results of the T-VEC/pembrolizumab combination in patients with recurrent or metastatic squamous cell carcinoma of the head and neck (HNSCC) [61]. Thirty-six participants were enrolled in the phase Ib part, and after a median follow-up of 5.8 months, one T-VEC-related DLT of fatal arterial hemorrhage was reported, but no fatal TRAEs were reported. The results showed that 55.6% and 58.3% of patients experienced AEs related to T-VEC and to pembrolizumab, respectively. Five (13.9%) patients confirmed PR and ten (27.8%) patients were non-evaluable for response due to early death. mPFS and mOS were 3.0 months and 5.8 months, respectively. This study provided reassurance as to the safe profile of the T-VEC/pembrolizumab combination in patients with HNSCC and noted a similar efficacy to that of pembrolizumab alone, as recorded in other HNSCC studies. The phase III part of this study was not further pursued.

### 2.2. HF-10

HF-10 is a natural clonal derivative in vitro-passaged laboratory strain of HSV-1, with spontaneous mutations but without the insertion of any foreign genes [62]. Due to some genomic deletions, HF-10 lacks the expression of functional UL43, UL49.5, UL55, UL56, and LAT genes [15,63]. These genetic differentiations were found in vitro to reduce the neuroinvasiveness of HSV without affecting viral replication, although the exact mechanisms are not clear [64,65]. Indeed, it has been shown that HF-10 is unable to invade the central nervous system and does not cause any neurological symptoms in mice when inoculated into the peripheral tissues [66]. It was initially tested in mice with disseminated peritoneal neoplasms [67]. In this immunocompetent animal model, the survival time of mice treated with HF10 was longer than that of mice treated with an avirulent, ribonucleotide reductase-deficient HSV-1, hrR3, that was used as a control. The oncolytic effect of HF10 was more potent than that of hrR3 and all of the surviving mice acquired resistance to rechallenge with tumor cell injection [67]. In vitro, HF10 was found to induce syncytia formation, whereas hrR3 formed rounded CPE. These results support the notion that HF-10 can exhibit a specific antitumor immune response [67]. In clinical phasing, only few studies have been completed for skin cancers, with one phase II multicenter trial presenting its results for the combination of HF-10 plus ipilimumab in advanced melanoma patients [68]. Of 46 patients enrolled and treated, 37% had ≥ grade 3 AEs, the majority due to IPI. Most HF-10-related AEs were ≤ grade 2, similar to HF10 monotherapy. HF10-related ≥ grade 3 AEs (*n* = 3) were embolism, lymphedema, diarrhea, hypoglycemia, and groin pain. Of the 44 efficacy-evaluable patients per irRC, ORR at 24 weeks was 41% (CR% = 16%), mPFS was 19 months and mOS was 21.8 months [68]. The authors concluded that the combination HF10 and ipilimumab demonstrated a favorable benefit/risk profile and encouraging antitumor activity in patients with stage IIIB, IIIC, or IV unresectable or metastatic melanoma. Currently, HF10 is also being tested in combination with nivolumab as a neoadjuvant therapy in potentially resectable stage IIIB, IIIC, or IVM1a melanoma (NCT03259425).

### 2.3. RP-1

RP-1 was introduced as a more potent alternative of T-VEC. RP-1 is created using a different strain of HSV-1, which was selected due to its increased cytotoxicity against cancer cells [69]. The selected virus was equipped with all the transgenic alterations that characterized T-VEC, with the addition of two more genes; GALV-GP-R−, a codon-optimized version of a potent fusogenic membrane glycoprotein (GP) from gibbon ape leukemia virus (GALV) and a gene expressing anti-CTLA-4 or immune costimulatory pathway-activating ligands. These alterations further enhanced the anticancer activity of the modified virus. Indeed, when it was tested against mouse lymphoma (A20 murine lymphoma model), as well as human lung and breast cancer cells (A549/MDA-MB-231), it exhibited increased efficacy, especially when combined with anti-PD-1 inhibition [69]. In clinical practice, RP-1 is tested either as a single agent (NCT04349436) or in combination with cemiplimab in advanced CSCC (NCT04050436), in order to maximize its efficacy. A phase I/II study (NCT03767348) is also underway examining the administration of RP-1 alone versus its combination with PD-1 blocker in patients with solid tumors, in general. The enrollment of this trial is expected to reach 300 participants.

## 3. Other Investigational OVs (Adenoviruses, Rhinoviruses, Coxsackieviruses, etc.)

Beyond herpesviruses, many other types of viruses are currently being tested as oncolytic vectors, and these have also exhibited some favorable characteristics (Table 2).

Adenoviruses (i) are immunogenic by their nature, (ii) are commonly used as vectors for gene transmissions, (iii) are easy to be produced in large quantities, and (iv) are well tolerated by human subjects. In skin cancers, these oncolytic adenoviral agents have been studied only using animal models or in early-phase trials. In 2021, Havunen et al. presented a novel oncolytic adenovirus, TILT-123, that expresses two potent cytokines, TNFa and IL-2, to stimulate the participation of T cells in the TME [23]. Its safety and biodistribution were initially studied in rodents showing well-tolerance, either as monotherapy and or in combination with an anti-PD-1 ICI. Treatment with TILT-123 induced acute changes in circulating immune cells, but cellular subpopulations returned to normal by the middle of the treatment period. TILT-123 was rapidly cleared from healthy tissues, and it did not cause damage to vital organs [23]. These results support the initiation of an open-label, dose-escalation, phase I clinical trial (NCT04217473) in refractory or recurrent stage III/IV melanoma patients, which cannot be treated with curative intent with available therapies and which are eligible for TIL therapy. According to the study design presented at the ESMO 2021 meeting, patients with at least one biopsiable/operable tumor for TIL generation and another injectable lesion for intratumoral TILT-123 administration would receive TILT-123 intravenously and intratumorally, as well as TIL therapy without pre- or post-conditioning [70]. Interim safety, efficacy, biological, immunological, and biosafety data are expected. ONCOS-102 is another oncolytic adenovirus armed with human GM-CSF and an Ad5/3 chimeric capsid. It was tested in four human melanoma cell lines, A375, A2058, SK-Mel-2, and SK-Mel-28, in combination with pembrolizumab [25]. Humanized mice engrafted with A2058 melanoma cells showed significant tumor volume reductions after ONCOS-102 treatment. The combination of pembrolizumab with ONCOS-102 reduced tumor volume to an even greater extent, whereas pembrolizumab did not show any therapeutic benefit by itself [25]. Weight loss and the development of metastasis were not significantly affected by any treatment. These data provided the scientific rationale for the phase I trial (NCT03003676) on the combination of ONCOS-102 and pembrolizumab for the treatment of anti-PD-1 refractory melanoma. This trial was divided into two parts, testing two different dosing schedules. In the first part, nine patients were given three intratumoral ONCOS-102 injections the first week, followed by treatment with pembrolizumab every third week up to 24 weeks. In the second part, 12 ICPI patients with more advanced disease than those in part 1 and who had shown disease progression in prior treatment with anti–PD-1 were enrolled in an extended dosing regimen of 12 intratumoral ONCOS-102 injections. During the first 2 weeks, patients received four injections, followed by the concomitant administration of ONCOS-102 and pembrolizumab from week 3 and every third week up to 24 weeks. Both parts showed favorable tolerability profiles with no safety concerns. In total, investigators detected that 35% of patients with PD-1 inhibitor–refractory melanoma ORR had tumor responses with the ONCOS-102 and pembrolizumab combination. As announced by Targovax, the company responsible for this agent, in a press release this summer, the FDA granted the fast-track designation to ONCOS-102 for the treatment of patients with PD-1 inhibitor–refractory advanced melanoma.

Among different adenoviruses, evidence of activity upon systemic administration is limited. The preclinical efficacy of a single intravenous administration of another oncolytic adenovirus type 5, responsive to the retinoblastoma pathway, which is commonly deregulated in tumors, led to a dose-escalation phase I trial in metastatic melanoma patients. The results of 14 patients treated with a single infusion of a dose of 1 × 10^11^ up to 1 × 10^13^ viral particles showed that ICOVIR5 was able to reach melanoma metastases upon a single intravenous administration, according to biopsies of lesions, but failed to induce tumor regressions [24].

An open-label phase I trial examined the intratumoral injection of a non-neurovirulent rhinovirus:poliovirus chimera (PVSRIPO) in patients with refractory melanoma stage III_B_-IV that showed disease progression on anti-PD-1 and BRAF/MEK inhibitors, if BRAF mutant. The injections of PVSRIPO were safe, with no serious AEs or DLTs. All AEs were grade 1 or 2 (pruritus grade 1 was the most common, at 58%); all but two PVSRIPO-TRAEs were localized to the injected or adjacent lesions (*n* = 1 hot flash grade 1, *n* = 1 fatigue grade 1). Twelve patients with lesion burdens (67% patients > 5 lesions) in four cohorts received a total of one, two, or three injections of PVSRIPO, with 21 days between injections. Four of 12 patients (33%) achieved an ORR per immune-related RECIST, and all had received three injections (four responders in the six patients who received three injections, 67%). In the four patients with in-transit disease, a pCR was observed in two of them (50%). Although the enrolled patients had already experienced progression in an ICI-based therapy, 92% of them (11/12) could be retreated with an ICI and 50% of them (6/12) remained without progression after the completion of intratumoral PVSRIPO administration at a median follow-up time of 18 months, with pCR observed in injected as well as non-injected lesions in select patients [26].

Coxsackievirus A21 (CAVATAK) is a naturally selected RNA OV without gene alterations, which preferentially infects cancer cells with an increased level of ICAM-1 receptors on their surface, leading to tumor cell lysis [71]. It has demonstrated activity against in vitro and in vivo melanoma cell lines and xenografts [72,73]. A phase Ib MITCI study evaluated the safety and efficacy of the combination of CAVATAK and ipilimumab in patients with treated or untreated unresectable stage III_C_-IV_M1c_ melanoma [27]. No DLTs were reported. The combination had minimal toxicity, with only one grade ≥3 ipilimumab-related fatigue. Of the 18 patients evaluable for response assessment, the confirmed ORR was 50% (9/18), 60% (6/10) in patients who were naive to ICIs and 38% (3/8) in those who had been exposed to ICIs. Notably, in patients with stage IV_M1c_ disease, ORR was 57.1% (4/7 patients). Responses were seen in both injected and uninjected lesions, as well as in patients with progression after immunotherapy [27]. The phase II, multicenter, open-label CALM study administered intralesionally CAVATAK in 57 patients with unresectable stage III_C_–IV_M1c_ melanoma [28]. The six-month PFS% was 36.8% (21 of 57 evaluable patients), which met the study’s primary endpoint. The ORR and the rate of CR or PR lasting ≥ 6 months per immune-related RECIST were 28.1% and 19.3%, respectively. No grade 3/4 AEs were reported [28]. At the AACR 2021 meeting, the preliminary results of the phase I CAPRA trial (NCT02565992) evaluating CAVATAK with pembrolizumab in patients with advanced melanoma showed manageable safety and promising efficacy [74]. In the 36 enrolled patients, no DLTs occurred and grade 3–5 TRAEs were reported in 14%, with treatment-related SAEs in three patients. ORR was 47% (CR, 22%; PR, 25%). Baseline tumor analysis showed that responses may not be associated with an inflamed TME since no differences in PD-L1 expression were found between responders and nonresponders; and lower levels of CD3^+^CD8^−^ infiltrates were detected in responders [75]. This combination is currently being studied in the neoadjuvant setting in patients with stage III melanoma (KEYMAKER-U02).

The reovirus serotype 3-Dearing strain is another OV that has been tested in patients with metastatic melanoma. It is a naturally occurring, ubiquitous, nonenveloped double-stranded RNA virus [76] that has been administered intravenously at a dose of 3 × 10^10^ TCID50 on days 1–5 of each 28-day cycle in a phase II trial in patients with metastatic melanoma. For the 21 enrolled patients, treatment was well tolerated, without any dose reductions having to be implemented. Viral replication was demonstrated in post-treatment biopsy samples but no efficacy could be established. No objective responses were observed and only one patient confirmed a 75–90% tumor necrosis, consistent with treatment effects after metastasectomy [76]. mPFS and mOS were 45 days and 165 days, respectively [76]. Based on preclinical data showing synergy with conventional chemotherapeutic compounds, a phase II combination trial in metastatic melanoma patients is ongoing.

Finally, the older example of an uncompleted approach to oncolytic virotherapy remains in regard to the development of Rigvir. Rigvir is an OV that belongs to the Picornaviridae family, the *Enterovirus* genus, the ECHO (Enteric Cytopathogenic Human Orphan) group, type 7, and was not genetically modified but selected because of its natural selectivity to melanoma cells [30,77]. In the 1950s and 1960s, it was registered for the prevention of melanoma relapse after radical surgery in Latvia, Georgia, Armenia, and Uzbekistan, but never achieved widespread use [29,30]. None of the Rigvir studies were randomized or double-blinded, and the control groups included historical controls. Studies outside Latvia were initiated in the early 1990s but never completed the trials due to political changes. In pre-registration efficacy studies, more than 540 patients with resected melanoma (mainly stage I–III and about 40 with stage IV) were treated with Rigvir for 3 years after surgery, and 3-and 5-year OS% results appeared to be increased for the Rigvir-treated patients [29]. The control group was treated with Corynebacterium parvum, splenin, zymosan, and levamisole [29]. In retrospective case studies, Rigvir-treated stage II melanoma patients showed a 6.67-fold decreased risk of disease progression in comparison to those who had remained in follow-up according to the standard guidelines then, and stage IB and stage II melanoma patients who had received Rigvir therapy exhibited 4.39–6.57-fold lower mortality [29]. However, if there is any continuation in this OV, it should be updated by more recent and randomized trials [30,77].

## 4. Reasons of Failure, Limitations, and Future Perspectives

With a critical view on the abovementioned evidence, we should conclude that the utilization of OVs in the treatment of skin cancer has shown more promises than indications up until now. The encouraging preclinical findings regarding OV-based regimens were merely replicated in early human studies and could not show any superiority against the standard of care at the phase III level of clinical testing. In fact, looking through the recent phase III trials on oncolytic virotherapy, the PHOCUS trial [78] comparing the combination of sorafenib and Pexa-Vec (a genetically engineered vaccinia poxvirus, JX-594) against sorafenib alone was terminated early after an interim futility analysis in patients with advanced liver cancer; similarly, the MASTERKEY-265/KEYNOTE-034 study [22] of T-VEC plus pembrolizumab versus pembrolizumab alone failed to meet its PFS primary endpoint, despite the promising phase Ib results of this combination in the same setting. In advanced melanoma, this was not the first time that the encouraging results from early clinical studies have not translated into survival benefits in a phase III trial. A similar story was seen with epacadostat plus pembrolizumab in the ECHO-301/KEYNOTE-252 study [79], with no survival benefits shown over pembrolizumab alone. There are many limitations that could have led to these discrepancies. As the researchers of PHOCUS trial characteristically admitted, “the immune system of rodents is much stronger than ours and preclinical models on immunotherapies are not always predictive of what will happen in humans”. In addition, the designations, the subject population (e.g., eligibility criteria and baseline characteristics), the statistical analyses performed, the primary and secondary endpoints, and even the assessment of the response in early clinical trials varied considerably among studies, making the generalization of results difficult. Furthermore, when a reasonably low number of patients displays positive phase II results, the tested OV-based regimens may be upgraded to the phase III level faster than they should be. In the case of combinatorial regimens, Andtbacka et al. noted that even the timing of the different components (lead-in, simultaneity, etc.) may affect the clinical outcomes [80]. Moreover, OV-based regimens have mainly been studied in pre-treated (and sometimes in heavily pretreated) patients with skin cancers, and therefore previously administered schemes may have altered the TME, influencing the observed result [46]. The phase III studies in patients with skin cancer usually include a heterogeneous sample of participants but not one that is large enough to allow the identification of specific parameters that may favor the oncolytic virotherapy. Even the relatively high exposure and acquired immunity of the general population against herpesviruses may have a negative effect on the efficacy of any HSV-1 derived OV [81,82]. However, this fact has been taken into account in preclinical studies in which the mice used were immunized against HSV-1 [75]. In the end, we can only speculate on the myriad potential reasons for the failures of phase III studies on OVs, but a future biomarker analysis may prove more informative in identifying those patients who are more likely to benefit from OV-based combinations.

## 5. Conclusions

While recovering from an unforeseen viral pandemic, we are at the timepoint of realizing the broad perspectives of integrating oncolytic viruses in the anticancer armamentarium. The discrepancy between the recent negative phase III results of the T-VEC/pembrolizumab combination and the promising data of T-VEC and other OVs in preclinical and early clinical levels demonstrates that we still have a lot of work to do in oncolytic virotherapy for skin cancers. Instead of examining the multiple reasons for not reaching the desirable endpoints, it would be more useful for ongoing credible attempts to become more focused on specific patients with melanoma or NMSC that could benefit more from an OV-based regimen. Acknowledging the lack of strong data on the hypotheses currently under investigation, an OV/ICI combination could be proven to be (i) an effective neoadjuvant option in patients with potentially resectable melanoma with high T-cell infiltrates at the initial biopsy, (ii) a reasonable approach to overcome resistance in patients with melanoma relapse after a long DoR in previous ICI, or (iii) an intensified therapeutic strategy in patients with advanced CSCC who are not eligible to be resected or radiated. Data from upcoming studies are expected to further improve our understanding of OVs, and to enable us to incorporate more precisely viral agents in the ever-growing field of oncological therapies.

## Figures and Tables

**Table 1:** Summary of characteristics of studied oncolytic herpesviruses. Abbreviations: HSV-1 = herpes simplex virus type 1. GALV-GP-R−: A codon-optimized version of a potent fusion membrane glycoprotein (GP) from gibbon ape leukemia virus (GALV). * anti-mouse CTLA-4 antibody-like molecule or mouse CD40L, mouse OX40L or mouse 4-1BBL.

T-VEC	RP1	HF-10
Selection of HSV-1 JS1 strain enhances selective targeting of tumor cells	Selection of HSV-1 RH018 strain offers increased cytotoxicity against tumor cells	Deletion in the Bam HI-B fragment
ICP34.5 gene deletion permits viral replication in tumor cells by attenuating the natural neurovirulence of the virus	ICP34.5 gene deletion permits viral replication in tumor cells by attenuating the natural neurovirulence of the virus	Non-expression of UL56 reduces neurovirulence of HSV without affecting viral replication in vitro
ICP47 gene deletion inhibits suppression of antigen presentation and upregulates HSV1 US11 gene	ICP47 gene deletion inhibits suppression of antigen presentation and upregulates HSV US11 gene	Reduced expression of UL43, UL49.5, UL55, and LAT reduces neurovirulence and enhances cell killing
HSV-1 US11 gene augments viral replication in tumor cells without impairing tumor selectivity	HSV US11 gene augments viral replication in tumor cells without impairing tumor selectivity	Increased expression of UL53 and UL54
	Expression of GALV-GP-R− * enhances systemic killing of tumor cells	
GM-CSF cassette initiates systemic immune response against tumor	GM-CSF cassette initiates systemic immune response against tumor	
	Expression of anti-CTLA-4 or immune co-stimulatory pathway activating ligands * further enhances systemic immune response	

**Table 2:** Main clinical trials on OV regimens in melanoma patients and their reported findings.

Author, Year Study Name (NCT#)	Phase (Status)	Therapy (Combination)	N	Stage of Melanoma Disease	ORR (%)	Main Outcomes (DoR, PFS, etc. in Months)	TRAE (Most Common Grade 3–4)
Hu JC et al., 2006	I (Completed)	T-VEC	30	Different metastatic tumors, including melanoma	N/A	N/A	Pyrexia, local inflammation, site erythema
Andtbacka RH et al., 2019 OPTiM (NCT00769704, EudraCT 2008-006140-20)	III (Completed)	T-VEC vs. GM-CSF	437	IIIB-IV	31.5 vs. 6.4	mDoR = not reached vs. 2.8 months	Cellulitis, tumor pain, vomiting, fatigue
(NCT01368276)	III (Completed)	T-VEC vs. GM-CSF	31	IIIB-IV	57.1 vs. 100	Extended safety study for eligible patients of NCT00769704	Cardiac disorders, vascular disorders, respiratory disorders, renal failure
Senzer et al., 2009 (NCT00289016)	II (Completed)	T-VEC	50	ΙΙΙC-IV	26	mDoR = 7.4 months (223 days)	Pain, fatigue, dyspnea
(NCT02574260)	II (Completed)	T-VEC	3	IIIB-IV	N/A	Participants who had received the maximum 24 treatments under NCT00289016 and met the inclusion and exclusion criteria were eligible to enroll	No grade 3–4 TRAEs
Andtbacka RH et al., 2019 (NCT02014441)	II (Completed)	T-VEC	61	IIIB-IVM1c	35	mDoR = not reached	Pyrexia, delirium
Puzanov I et al., 2016 (NCT01740297)	Ib (Completed)	T-VEC + Ipilimumab	19	IIIC-IV	50	mDoR = not reached mPFS = not reached 18 month-PFS% = 50%	Nausea, lipase and amylase increase (IPI-related)
Chesney J et al., 2019 (NCT01740297)	II (Completed)	T-VEC + Ipilimumab vs. Ipilimumab	198	IIIC-IV	36.7 vs. 16	mDoR = not reached mPFS = 13.5 vs. 4.5 months	Colitis, diarrhea, influenza-like symptoms, lymphopenia
Malvehy J et al., 2021 (TVEC-325) (NCT02366195)	II (Completed)	T-VEC	112	IIIB-IVM1c	32	mDoR = not reached mTTF = 8.1 months	Metastatic melanoma, metastases to central nervous system, general physical deterioration, pyrexia, back pain
Tulokas SKA et al., 2021 (NCT03555032 NCT02094391 NCT03685890 NCT03555032)	I/II (Completed)	Ipilimumab vs. Nivolumab vs. T-VEC	60	IIIB-IV	77	mPFS = 6.1	Cellulitis, gastrointestinal disorders, pyrexia/influenza, pain/post-operative wound infection
(NCT03003676)	I (Completed)	ONCOS-102 + cyclophosphamide + pembrolizumab	21	Relapsed melanoma after prior PD-1 blockade	37.5 for part 1, 33.3 for part 2	N/A	Enterocolitis, pyrexia, syncope, cough, dyspnea
Robert L Ferris et al., 2015 (NCT01017185)	I (Completed)	HF10 + Ipilimumab	28	Various skin cancers, including melanoma	N/A	N/A	N/A
Andtbacka R.H.I et al., 2017 (NCT02272855)	II (Completed)	HF10 + Ipilimumab	46	IIIB-IV	41	mPFS = 19 months	Embolism, lymphedema, diarrhea, hypoglycemia, and groin pain
Yokota K et al., 2019 (NCT03153085)	II (Completed)	HF10 + Ipilimumab	28	IIIB-IV	BORR = 11.1%	DCR = 55.6%	Grade 3 TRAEs = 35.7%
Dummer R et al., 2021 (NCT02211131)	II (Active)	Neoadjuvant T-VEC + surgical resection vs. immediate surgical resection	150	IIIB-IVM1a	Lesion ORR = 26.3 vs 3.9 (T-VEC arm only, injected vs. uninjected lesions	2-year RFS% = 29.5% vs. 16.5%	Cellulitis, pyrexia, cholecystitis
Yamazaki N et al., 2018 (NCT03064763)	I (Active, not recruiting)	T-VEC	18	IIIB-IV	N/A	N/A	Infectious enteritis, worsening of benign prostatic hyperplasia, epiglottitis, pneumonia
Long G et al., 2019 (NCT02263508)	Ib (Completed)	T-VEC + pembrolizumab	21	IIIB-IVM1c	62	mDoR = not reached mPFS = not Reached 4-year PFS% = 55.9%	Fatigue, pyrexia, chills
Ribas A et al., 2021 MASTERKEY-265/KEYNOTE-034 (NCT02263508)	III (Completed)	T-VEC + pembrolizumab vs. Placebo + pembrolizumab	692	IIIB-IVM1c	48.6 vs. 41.3	mDoR = 43.7 vs. not reached mPFS = 14.3 vs. 8.5 months	Fatigue, pyrexia, chills
NIVEC (NCT04330430)	II (Recruiting)	Neoadjuvant T-VEC+nivolumab for 8 weeks	24	IIIB-IVM1a	N/A	N/A	N/A
Beasley GM et al., 2021 (NCT03712358)	I (Active, not recruiting)	PVSRIPO	18	IIIB-IV	33	18-month PFS% = 50%	No grade 3–4 TRAEs
Wang X et al., 2021 (NCT04197882)	Ib (Active, not recruiting)	OrienX010 + toripalimab	33	IIIB-IVM1a	N/A	N/A	Alanine aminotransferase increase, wound infections
Guo J et al., 2021 (NCT04206358)	Ib (Recruiting)	OrienX010 + JS001	30	IV (M1c)	13.3	mPFS = not reached	No grade 3–4 TRAEs
(NCT04125719)	I (Withdrawn and planned to be resubmitted)	PVSRIPO + nivolumab	0	IIIB-IV	N/A	N/A	N/A
(NCT04577807)	II (Recruiting)	PVSRIPO vs. PVSRIPO + anti-PD-1 ICI	56	Advanced melanoma refractory to PD-1 blockade	N/A	N/A	N/A
(NCT03259425)	II (Terminated, DSMC recommendation)	HF10 + nivolumab	7	IIIB-IVM1a	N/A	N/A	Anemia, skin and subcutaneous tissue disorders
(NCT04427306)	II (Recruiting)	T-VEC	62	High-risk, resectable melanoma	N/A	N/A	N/A
(NCT03842943)	II (Recruiting)	T-VEC + pembrolizumab	28	III	N/A	N/A	N/A
(NCT02965716)	II (Active)	T-VEC + pembrolizumab	47	IIIA-IV	N/A	N/A	N/A
(NCT04068181)	II (Active)	T-VEC + pembrolizumab	72	IIIB-IVM1d	N/A	N/A	N/A
(NCT02297529)	IIIB (Recruiting)	T-VEC	-	IIIB-IVM1c	N/A	N/A	N/A
(NCT03747744)	I (Active)	CD1c (BDCA-1) + myDC + T-VEC	18	Advanced/metastatic melanoma	N/A	N/A	N/A
Thomas S et al., 2019 (NCT03767348)	II (Recruiting)	RP1 vs. RP1 + nivolumab	300	Various solid tumors, including melanoma	N/A	N/A	N/A
(NCT04123470)	I/II (Recruiting)	delolimogene mupadenorepvec + atezolizumab	35	Metastatic melanoma	N/A	N/A	N/A
Havunen R et al., 2021 (NCT04217473)	I (Recruiting)	TNFalpha + TILT-123	15	Refractory/recurrent stage III-IV melanoma	N/A	N/A	N/A
(NCT02819843)	II (Active)	T-VEC + Hypofractionated Radiotherapy vs. T-VEC	19	Various solid tumors, including melanoma	N/A	N/A	N/A
Garcia et al., 2019 (NCT01864759)	I (Completed)	ICOVIR-5	14	Uveal or cutaneous metastatic melanoma	N/A	N/A	Transaminase increase, asthenia, edema
Curti BD et al., 2017 (NCT02307149)	I (Completed)	CAVATAK + ipilimumab	18	IIIB-IV	BORR = 38%	DCR = 88%	Fatigue (IPI-related)
Andtbacka RH et al., 2015 (NCT01227551)	II (Completed)	CAVATAK	57	IIIC–IVM1c	28.1	6-month PFS% = 38.6%	No grade 3/4 TRAEs
Silk et al., 2021 (NCT02565992)	I (Completed)	CAVATAK + pembrolizumab	36	IIIB-IV	47	mDoR = not reached mPFS = 11.9	Autoimmune encephalitis, septic shock, keratoacanthoma, autoimmune hepatitis

Abbreviations: N/A = not available; N = number of patients; ORR = objective response rate; DoR = duration of response; PFS = progression free survival; DCR = disease control rate; TTF = time to treatment failure; RFS = regression free survival; BORR = best overall response rate; TRAE = treatment-related adverse events; T-VEC = talimogene laherparepvec; GM-CSF = granulocyte-macrophage colony-stimulating factor; DSMC = Data and Safety Monitoring Committee; ICI = immune checkpoint inhibitor.

**Table 3:** Main clinical trials on OV regimens in non-melanoma skin cancers and their reported findings.

Author, Study Name (NCT#)	Phase (Status)	Therapy (Combination)	N	Study Population	ORR (%)	Main Outcomes (DoR, PFS, etc. in Months	TRAE (Most Common Grade 3–4)
(NCT03458117)	I (Recruiting)	T-VEC	20	SCC	N/A	N/A	N/A
(NCT04163952)	I (Recruiting)	T-VEC + Panitumumab	30	SCC	N/A	N/A	N/A
(NCT04050436)	II (Recruiting)	RP1 + Cemiplimab vs. Cemiplimab	180	SCC	N/A	N/A	N/A
(NCT03714828)	II (Recruiting)	T-VEC	28	SCC	N/A	N/A	N/A
(NCT01161498)	III (Terminated)	T-VEC + Radiation + Cisplatin vs. Radiation + Cisplatin	5	HNSCC	N/A	N/A	Lung infection, urinary tract infection, hyperglycemia, malignant neoplasm progression, acute renal failure, pleural effusion
(NCT04349436)	Ιb (recruiting)	RP1	30	SCC	N/A	N/A	N/A
Harrington et al., 2021 (NCT02626000)	Ιb (Completed)	T-VEC + pembrolizumab	36	Recurrent or metastatic HNSCC	16.7	mDoR = 45.9 months mPFS = 3 months	Pyrexia, arterial hemorrhage, chills, mucosal hemorrhage
(NCT03458117)	I (Recruiting)	T-VEC	20	BCC	N/A	N/A	N/A
(NCT03458117)	I (Recruiting)	T-VEC	20	MCC	N/A	N/A	N/A
(NCT02819843)	II (Active, not recruiting)	T-VEC	19	MCC	N/A	N/A	N/A
(NCT03921073)	II (Active, not recruiting)	T-VEC	5	Angiosarcoma of the skin	N/A	N/A	N/A
Kelly CM et al., 2020 (NCT03069378)	II (Recruiting)	T-VEC + pembrolizumab	20	Locally advanced/ metastatic sarcoma	35	mDoR = 14 months (56.1 weeks) mPFS = 4.3 months (17.1 weeks)	Pneumonitis, anemia, fever, hypophosphatemia

Abbreviations: N/A = not available; N = number of patients; ORR = objective response rate; DoR = duration of response; PFS = progression free survival; TRAE = treatment-related adverse events; T-VEC = talimogene laherparepvec; SCC = squamous cell carcinoma; BCC = basal cell carcinoma; MCC = Merkel cell carcinoma; HNSCC = head and neck SCC.

## Data Availability

Data supporting the recommendations of this article are included within the reference list. Please contact the corresponding author for any further data request or supplementary information.

## References

[1] Kasakovski D., Skrygan M., Gambichler T., Susok L. (2021). Advances in Targeting Cutaneous Melanoma. Cancers.

[2] Paulson K.G., Gupta D., Kim T.S., Veatch J.R., Byrd D.R., Bhatia S., Wojcik K., Chapuis A.G., Thompson J.A., Madeleine M.M. (2020). Age-Specific Incidence of Melanoma in the United States. JAMA Dermatol..

[3] Apalla Z., Nashan D., Weller R.B., Castellsague X. (2017). Skin Cancer: Epidemiology, Disease Burden, Pathophysiology, Diagnosis, and Therapeutic Approaches. Dermatol. Ther..

[4] Ascierto P.A., Garbe C. (2020). Updates and new perspectives in nonmelanoma skin cancer therapy: Highlights from ‘Immunotherapy Bridge’. Immunotherapy.

[5] Keilholz U., Ascierto P.A., Dummer R., Robert C., Lorigan P., van Akkooi A., Arance A., Blank C.U., Chiarion Sileni V., Donia M. (2020). ESMO consensus conference recommendations on the management of metastatic melanoma: Under the auspices of the ESMO Guidelines Committee. Ann. Oncol..

[6] Chalmers Z.R., Connelly C.F., Fabrizio D., Gay L., Ali S.M., Ennis R., Schrock A., Campbell B., Shlien A., Chmielecki J. (2017). Analysis of 100,000 human cancer genomes reveals the landscape of tumor mutational burden. Genome Med..

[7] Cives M., Mannavola F., Lospalluti L., Sergi M.C., Cazzato G., Filoni E., Cavallo F., Giudice G., Stucci L.S., Porta C. (2020). Non-Melanoma Skin Cancers: Biological and Clinical Features. Int. J. Mol. Sci..

[8] Stonesifer C.J., Djavid A.R., Grimes J.M., Khaleel A.E., Soliman Y.S., Maisel-Campbell A., Garcia-Saleem T.J., Geskin L.J., Carvajal R.D. (2021). Immune Checkpoint Inhibition in Non-Melanoma Skin Cancer: A Review of Current Evidence. Front. Oncol..

[9] Wolchok J.D., Chiarion-Sileni V., Gonzalez R., Grob J.-J., Rutkowski P., Lao C.D., Cowey C.L., Schadendorf D., Wagstaff J., Dummer R. (2021). CheckMate 067: 6.5-Year outcomes in patients (pts) with advanced melanoma. J. Clin. Oncol..

[10] Hughes B.G.M., Munoz-Couselo E., Mortier L., Bratland A., Gutzmer R., Roshdy O., Gonzalez Mendoza R., Schachter J., Arance A., Grange F. (2021). Pembrolizumab for locally advanced and recurrent/metastatic cutaneous squamous cell carcinoma (KEYNOTE-629 study): An open-label, nonrandomized, multicenter, phase II trial. Ann. Oncol..

[11] Migden M.R., Rischin D., Schmults C.D., Guminski A., Hauschild A., Lewis K.D., Chung C.H., Hernandez-Aya L., Lim A.M., Chang A.L.S. (2018). PD-1 Blockade with Cemiplimab in Advanced Cutaneous Squamous-Cell Carcinoma. N. Engl. J. Med..

[12] Tawbi H.A., Schadendorf D., Lipson E.J., Ascierto P.A., Matamala L., Castillo Gutiérrez E., Rutkowski P., Gogas H.J., Lao C.D., De Menezes J.J. (2022). Relatlimab and Nivolumab versus Nivolumab in Untreated Advanced Melanoma. N. Engl. J. Med..

[13] Kelly E., Russell S.J. (2007). History of oncolytic viruses: Genesis to genetic engineering. Mol. Ther..

[14] Moreno R. (2021). Mesenchymal stem cells and oncolytic viruses: Joining forces against cancer. J. Immunother. Cancer.

[15] Watanabe D., Goshima F. (2018). Oncolytic Virotherapy by HSV. Adv. Exp. Med. Biol..

[16] Liu B.L., Robinson M., Han Z.Q., Branston R.H., English C., Reay P., McGrath Y., Thomas S.K., Thornton M., Bullock P. (2003). ICP34.5 deleted herpes simplex virus with enhanced oncolytic, immune stimulating, and anti-tumour properties. Gene Ther..

[17] Gao P., Ding G., Wang L. (2021). The efficacy and safety of oncolytic viruses in the treatment of intermediate to advanced solid tumors: A systematic review and meta-analysis. Transl. Cancer Res..

[18] Rahman M.M., McFadden G. (2021). Oncolytic Viruses: Newest Frontier for Cancer Immunotherapy. Cancers.

[19] Hamid O., Ismail R., Puzanov I. (2020). Intratumoral Immunotherapy-Update 2019. Oncologist.

[20] Marabelle A., Tselikas L., de Baere T., Houot R. (2017). Intratumoral immunotherapy: Using the tumor as the remedy. Ann. Oncol..

[21] Lawler S.E., Speranza M.C., Cho C.F., Chiocca E.A. (2017). Oncolytic Viruses in Cancer Treatment: A Review. JAMA Oncol..

[22] Ribas A., Chesney J., Long G.V., Kirkwood J.M., Dummer R., Puzanov I., Hoeller C., Gajewski T.F., Gutzmer R., Rutkowski P. (2021). 1037O MASTERKEY-265: A phase III, randomized, placebo (Pbo)-controlled study of talimogene laherparepvec (T) plus pembrolizumab (P) for unresectable stage IIIB–IVM1c melanoma (MEL). Ann. Oncol..

[23] Havunen R., Kalliokoski R., Siurala M., Sorsa S., Santos J.M., Cervera-Carrascon V., Anttila M., Hemminki A. (2021). Cytokine-Coding Oncolytic Adenovirus TILT-123 Is Safe, Selective, and Effective as a Single Agent and in Combination with Immune Checkpoint Inhibitor Anti-PD-1. Cells.

[24] Garcia M., Moreno R., Gil-Martin M., Cascallo M., de Olza M.O., Cuadra C., Piulats J.M., Navarro V., Domenech M., Alemany R. (2019). A Phase 1 Trial of Oncolytic Adenovirus ICOVIR-5 Administered Intravenously to Cutaneous and Uveal Melanoma Patients. Hum. Gene Ther..

[25] Kuryk L., Moller A.W., Jaderberg M. (2019). Combination of immunogenic oncolytic adenovirus ONCOS-102 with anti-PD-1 pembrolizumab exhibits synergistic antitumor effect in humanized A2058 melanoma huNOG mouse model. Oncoimmunology.

[26] Beasley G.M., Nair S.K., Farrow N.E., Landa K., Selim M.A., Wiggs C.A., Jung S.H., Bigner D.D., True Kelly A., Gromeier M. (2021). Phase I trial of intratumoral PVSRIPO in patients with unresectable, treatment-refractory melanoma. J Immunother. Cancer.

[27] Curti B.D., Richards J.M., Hallmeyer S., Faries M.B., Andtbacka R.H.I., Daniels G.A., Grose M., Shafren D. (2017). Activity of a novel immunotherapy combination of intralesional Coxsackievirus A21 and systemic ipilimumab in advanced melanoma patients previously treated with anti-PD1 blockade therapy. J. Clin. Oncol..

[28] Andtbacka R.H.I., Curti B.D., Kaufman H., Daniels G.A., Nemunaitis J.J., Spitler L.E., Hallmeyer S., Lutzky J., Schultz S.M., Whitman E.D. (2015). Final data from CALM: A phase II study of Coxsackievirus A21 (CVA21) oncolytic virus immunotherapy in patients with advanced melanoma. J. Clin. Oncol..

[29] Donina S., Strele I., Proboka G., Auzins J., Alberts P., Jonsson B., Venskus D., Muceniece A. (2015). Adapted ECHO-7 virus Rigvir immunotherapy (oncolytic virotherapy) prolongs survival in melanoma patients after surgical excision of the tumour in a retrospective study. Melanoma Res..

[30] Alberts P., Tilgase A., Rasa A., Bandere K., Venskus D. (2018). The advent of oncolytic virotherapy in oncology: The Rigvir(R) story. Eur. J. Pharmacol..

[31] McGeoch D.J., Dalrymple M.A., Davison A.J., Dolan A., Frame M.C., McNab D., Perry L.J., Scott J.E., Taylor P. (1988). The complete DNA sequence of the long unique region in the genome of herpes simplex virus type 1. J. Gen. Virol..

[32] Marconi P., Argnani R., Berto E., Epstein A.L., Manservigi R. (2008). HSV as a vector in vaccine development and gene therapy. Hum. Vaccin..

[33] Todo T. (2008). Oncolytic virus therapy using genetically engineered herpes simplex viruses. Front. Biosci..

[34] Fu X., Zhang X. (2002). Potent systemic antitumor activity from an oncolytic herpes simplex virus of syncytial phenotype. Cancer Res..

[35] Wang P.Y., Swain H.M., Kunkler A.L., Chen C.Y., Hutzen B.J., Arnold M.A., Streby K.A., Collins M.H., Dipasquale B., Stanek J.R. (2016). Neuroblastomas vary widely in their sensitivities to herpes simplex virotherapy unrelated to virus receptors and susceptibility. Gene Ther..

[36] Toda M., Martuza R.L., Rabkin S.D. (2000). Tumor growth inhibition by intratumoral inoculation of defective herpes simplex virus vectors expressing granulocyte-macrophage colony-stimulating factor. Mol. Ther..

[37] Kohlhapp F.J., Kaufman H.L. (2016). Molecular Pathways: Mechanism of Action for Talimogene Laherparepvec, a New Oncolytic Virus Immunotherapy. Clin. Cancer Res..

[38] Cooke K., Estrada J., Zhan J., Mitchell P., Bulliard Y., Beltran P.J. (2016). Abstract 2351: Development of a B16F10 cell line expressing mNectin1 to study the activity of OncoVEXmGM-CSF in murine syngeneic melanoma models. Cancer Res..

[39] Cooke K., Rottman J., Zhan J., Mitchell P., Ikotun O., Yerby B., Chong A., Glaus C., Moesta A.K., Pedro B. (2015). Oncovex MGM-CSF –mediated regression of contralateral (non-injected) tumors in the A20 murine lymphoma model does not involve direct viral oncolysis. J. Immunother. Cancer.

[40] Piasecki J., Tiep L., Zhou J., Beers C. (2013). Talilmogene Iaherparepvec generates systemic T-cell-mediated anti-tumor immunity. J. Immunother. Cancer.

[41] Moesta A.K., Cooke K., Piasecki J., Mitchell P., Rottman J.B., Fitzgerald K., Zhan J., Yang B., Le T., Belmontes B. (2017). Local Delivery of OncoVEXmGM-CSF Generates Systemic Antitumor Immune Responses Enhanced by Cytotoxic T-Lymphocyte–Associated Protein Blockade. Clin. Cancer Res..

[42] Hu J.C., Coffin R.S., Davis C.J., Graham N.J., Groves N., Guest P.J., Harrington K.J., James N.D., Love C.A., McNeish I. (2006). A phase I study of OncoVEXGM-CSF, a second-generation oncolytic herpes simplex virus expressing granulocyte macrophage colony-stimulating factor. Clin. Cancer Res..

[43] Yamazaki N., Koga H., Kojima T., Tsutsumida A., Namikawa K., Yi M., Mera K., Pickett-Gies C. (2018). Early safety from a phase I, multicenter, open-label, dose de-escalation study of talimogene laherparepvec (T-VEC) in Japanese patients (pts) with unresectable stage IIIB-IV melanoma (MEL). Ann. Oncol..

[44] Senzer N.N., Kaufman H.L., Amatruda T., Nemunaitis M., Reid T., Daniels G., Gonzalez R., Glaspy J., Whitman E., Harrington K. (2009). Phase II clinical trial of a granulocyte-macrophage colony-stimulating factor-encoding, second-generation oncolytic herpesvirus in patients with unresectable metastatic melanoma. J. Clin. Oncol..

[45] Kaufman H.L., Kim D.W., DeRaffele G., Mitcham J., Coffin R.S., Kim-Schulze S. (2010). Local and distant immunity induced by intralesional vaccination with an oncolytic herpes virus encoding GM-CSF in patients with stage IIIc and IV melanoma. Ann. Surg. Oncol..

[46] Malvehy J., Samoylenko I., Schadendorf D., Gutzmer R., Grob J.J., Sacco J.J., Gorski K.S., Anderson A., Pickett C.A., Liu K. (2021). Talimogene laherparepvec upregulates immune-cell populations in non-injected lesions: Findings from a phase II, multicenter, open-label study in patients with stage IIIB-IVM1c melanoma. J. Immunother. Cancer.

[47] Andtbacka R.H.I., Amatruda T., Nemunaitis J., Zager J.S., Walker J., Chesney J.A., Liu K., Hsu C.P., Pickett C.A., Mehnert J.M. (2019). Biodistribution, shedding, and transmissibility of the oncolytic virus talimogene laherparepvec in patients with melanoma. EBioMedicine.

[48] Andtbacka R.H.I., Collichio F., Harrington K.J., Middleton M.R., Downey G., hrling K., Kaufman H.L. (2019). Final analyses of OPTiM: A randomized phase III trial of talimogene laherparepvec versus granulocyte-macrophage colony-stimulating factor in unresectable stage III-IV melanoma. J. Immunother. Cancer.

[49] Gogas H., Samoylenko I., Schadendorf D., Gutzmer R., Grob J.J., Sacco J.J., Gorski K., Anderson A., Liu C., Malvehy J. (2018). Talimogene laherparepvec (T-VEC) treatment increases intratumoral effector T-cell and natural killer (NK) cell density in noninjected tumors in patients (pts) with stage IIIB–IVM1c melanoma: Evidence for systemic effects in a phase II, single-arm study. Ann. Oncol..

[50] Chesney J.A., Puzanov I., Collichio F., Singh P., Milhem M., Glaspy J., Hamid O., Ross M.I., Friedlander P., Garbe C. (2019). Talimogene laherparepvec (T-VEC) in combination (combo) with ipilimumab (ipi) versus ipi alone for advanced melanoma: 3-year landmark analysis of a randomized, open-label, phase II trial. Ann. Oncol..

[51] Hu-Lieskovan S., Moon J., Campos D., Grossmann K.F., Sosman J.A., Ryan C.W., Wu M., Ribas A. (2018). Reversing resistance to PD-1 blockade by combination of talimogene laherparepvec (T-VEC) with pembrolizumab (pembro) in advanced melanoma patients following progression on a prior PD-1 inhibitor: SWOG S1607 (NCT#02965716). J. Clin. Oncol..

[52] Long G., Dummer R., Johnson D., Michielin O., Martin-Algarra S., Treichel S., Chan E., Diede S., Ribas A. (2020). 429|Long-term analysis of MASTERKEY-265 phase 1b trial of talimogene laherparepvec (T-VEC) plus pembrolizumab in patients with unresectable stage IIIB-IVM1c melanoma. J. Immunother. Cancer.

[53] Dummer R., Gyorki D., Hyngstrom J., Berger A., Conry R., Demidov L., Chan E., Radcliffe H.-S., Faries M., Ross M. (2020). 432|3-year results of the phase 2 randomized trial for talimogene laherparepvec (T-VEC) neoadjuvant treatment plus surgery vs surgery in patients with resectable stage IIIB-IVM1a melanoma. J. Immunother. Cancer.

[54] Dummer R., Gyorki D.E., Hyngstrom J., Berger A.C., Conry R., Demidov L., Sharma A., Treichel S.A., Radcliffe H., Gorski K.S. (2021). Neoadjuvant talimogene laherparepvec plus surgery versus surgery alone for resectable stage IIIB-IVM1a melanoma: A randomized, open-label, phase 2 trial. Nat. Med..

[55] Tulokas S.K.A., Kohtamaki L.M., Makela S.P., Juteau S., Alback A., Vikatmaa P.J., Mattila K.E., Skytta T.K., Koivunen J.P., Tyynela-Korhonen K. (2021). Isolated limb perfusion with melphalan as treatment for regionally advanced melanoma of the limbs: Results of 60 patients treated in Finland during 2007–2018. Melanoma Res..

[56] Cui C., Wang X., Lian B., Ji Q., Zhou L., Chi Z., Si L., Sheng X., Kong Y., Yu J. (2022). OrienX010, an oncolytic virus, in patients with unresectable stage IIIC-IV melanoma: A phase Ib study. J. Immunother. Cancer.

[57] Wang X., Cui C., Si L., Li C., Dai J., Mao L., Bai X., Chi Z., Sheng X., Kong Y. (2021). A phase Ib clinical trial of neoadjuvant OrienX010, an oncolytic virus, in combination with toripalimab in patients with resectable stage IIIb to stage IVM1a acral melanoma. J. Clin. Oncol..

[58] Zhang L., Hao B., Geng Z., Geng Q. (2021). Toripalimab: The First Domestic Anti-Tumor PD-1 Antibody in China. Front. Immunol..

[59] Guo J., Cui C., Wang X., Lian B., Yin S., Cong Y., Chi Z., Si L., Sheng X., Tang B. (2021). A phase 1b clinical trial of anti-PD-1 ab (Toripalimab) plus intralesional injection of OrienX010 in stage melanoma with liver metastases. J. Clin. Oncol..

[60] Kelly C.M., Antonescu C.R., Bowler T., Munhoz R., Chi P., Dickson M.A., Gounder M.M., Keohan M.L., Movva S., Dholakia R. (2020). Objective Response Rate Among Patients with Locally Advanced or Metastatic Sarcoma Treated With Talimogene Laherparepvec in Combination With Pembrolizumab: A Phase 2 Clinical Trial. JAMA Oncol..

[61] Harrington K.J., Kong A., Mach N., Chesney J.A., Fernandez B.C., Rischin D., Cohen E.E.W., Radcliffe H.S., Gumuscu B., Cheng J. (2020). Talimogene Laherparepvec and Pembrolizumab in Recurrent or Metastatic Squamous Cell Carcinoma of the Head and Neck (MASTERKEY-232): A Multicenter, Phase 1b Study. Clin. Cancer Res..

[62] Eissa I.R., Naoe Y., Bustos-Villalobos I., Ichinose T., Tanaka M., Zhiwen W., Mukoyama N., Morimoto T., Miyajima N., Hitoki H. (2017). Genomic Signature of the Natural Oncolytic Herpes Simplex Virus HF10 and Its Therapeutic Role in Preclinical and Clinical Trials. Front. Oncol..

[63] Ushijima Y., Luo C., Goshima F., Yamauchi Y., Kimura H., Nishiyama Y. (2007). Determination and analysis of the DNA sequence of highly attenuated herpes simplex virus type 1 mutant HF10, a potential oncolytic virus. Microbes Infect..

[64] Koshizuka T., Kawaguchi Y., Nishiyama Y. (2005). Herpes simplex virus type 2 membrane protein UL56 associates with the kinesin motor protein KIF1A. J. Gen. Virol..

[65] Jones C., Inman M., Peng W., Henderson G., Doster A., Perng G.C., Angeletti A.K. (2005). The herpes simplex virus type 1 locus that encodes the latency-associated transcript enhances the frequency of encephalitis in male BALB/c mice. J. Virol..

[66] Nawa A., Luo C., Zhang L., Ushjima Y., Ishida D., Kamakura M., Fujimoto Y., Goshima F., Kikkawa F., Nishiyama Y. (2008). Non-engineered, naturally oncolytic herpes simplex virus HSV1 HF-10: Applications for cancer gene therapy. Curr. Gene Ther..

[67] Takakuwa H., Goshima F., Nozawa N., Yoshikawa T., Kimata H., Nakao A., Nawa A., Kurata T., Sata T., Nishiyama Y. (2003). Oncolytic viral therapy using a spontaneously generated herpes simplex virus type 1 variant for disseminated peritoneal tumor in immunocompetent mice. Arch. Virol..

[68] Andtbacka R.H.I., Ross M.I., Agarwala S.S., Taylor M.H., Vetto J.T., Neves R.I., Daud A., Khong H.T., Ungerleider R.S., Tanaka M. (2017). Final results of a phase II multicenter trial of HF10, a replication-competent HSV-1 oncolytic virus, and ipilimumab combination treatment in patients with stage IIIB-IV unresectable or metastatic melanoma. J. Clin. Oncol..

[69] Thomas S., Kuncheria L., Roulstone V., Kyula J.N., Mansfield D., Bommareddy P.K., Smith H., Kaufman H.L., Harrington K.J., Coffin R.S. (2019). Development of a new fusion-enhanced oncolytic immunotherapy platform based on herpes simplex virus type 1. J. Immunother. Cancer.

[70] Svane I.M., Santos J.M., Cervera-Carrascon V., Havunen R., Sorsa S., Ellebæk E., Monberg T., Donia M., Khammari A., Dréno B. (2021). 1032TiP A phase I, first-in-human, study of TILT-123, a tumor-selective oncolytic adenovirus encoding TNFa and IL-2, in participants with advanced melanoma receiving adoptive T-cell therapy with tumor-infiltrating lymphocytes. Ann. Oncol..

[71] Xiao C., Bator-Kelly C.M., Rieder E., Chipman P.R., Craig A., Kuhn R.J., Wimmer E., Rossmann M.G. (2005). The crystal structure of coxsackievirus A21 and its interaction with ICAM-1. Structure.

[72] Shafren D.R., Au G.G., Nguyen T., Newcombe N.G., Haley E.S., Beagley L., Johansson E.S., Hersey P., Barry R.D. (2004). Systemic therapy of malignant human melanoma tumors by a common cold-producing enterovirus, coxsackievirus a21. Clin. Cancer Res..

[73] Au G.G., Lindberg A.M., Barry R.D., Shafren D.R. (2005). Oncolysis of vascular malignant human melanoma tumors by Coxsackievirus A21. Int. J. Oncol..

[74] Silk A.W., O’Day S.J., Kaufman H.L., Bryan J., Norrell J.T., Imbergamo C., Portal D., Zambrano-Acosta E., Palmeri M., Fein S. (2021). Abstract CT139: Intratumoral oncolytic virus V937 in combination with pembrolizumab (pembro) in patients (pts) with advanced melanoma: Updated results from the phase 1b CAPRA study. Cancer Res..

[75] Chahlavi A., Rabkin S., Todo T., Sundaresan P., Martuza R. (1999). Effect of prior exposure to herpes simplex virus 1 on viral vector-mediated tumor therapy in immunocompetent mice. Gene Ther..

[76] Galanis E., Markovic S.N., Suman V.J., Nuovo G.J., Vile R.G., Kottke T.J., Nevala W.K., Thompson M.A., Lewis J.E., Rumilla K.M. (2012). Phase II trial of intravenous administration of Reolysin((R)) (Reovirus Serotype-3-dearing Strain) in patients with metastatic melanoma. Mol. Ther..

[77] Alberts P., Olmane E., Brokane L., Krastina Z., Romanovska M., Kupcs K., Isajevs S., Proboka G., Erdmanis R., Nazarovs J. (2016). Long-term treatment with the oncolytic ECHO-7 virus Rigvir of a melanoma stage IV M1c patient, a small cell lung cancer stage IIIA patient, and a histiocytic sarcoma stage IV patient-three case reports. APMIS.

[78] Abou-Alfa G.K., Galle P.R., Chao Y., Brown K.T., Heo J., Borad M.J., Luca A., Pelusio A., Agathon D., Lusky M. (2016). PHOCUS: A phase 3 randomized, open-label study comparing the oncolytic immunotherapy Pexa-Vec followed by sorafenib (SOR) vs SOR in patients with advanced hepatocellular carcinoma (HCC) without prior systemic therapy. J. Clin. Oncol..

[79] Long G.V., Dummer R., Hamid O., Gajewski T.F., Caglevic C., Dalle S., Arance A., Carlino M.S., Grob J.J., Kim T.M. (2019). Epacadostat plus pembrolizumab versus placebo plus pembrolizumab in patients with unresectable or metastatic melanoma (ECHO-301/KEYNOTE-252): A phase 3, randomised, double-blind study. Lancet Oncol..

[80] Andtbacka R.H., Kaufman H.L., Collichio F., Amatruda T., Senzer N., Chesney J., Delman K.A., Spitler L.E., Puzanov I., Agarwala S.S. (2015). Talimogene Laherparepvec Improves Durable Response Rate in Patients with Advanced Melanoma. J. Clin. Oncol..

[81] Uche I.K., Fowlkes N., Vu L., Watanabe T., Carossino M., Nabi R., Del Piero F., Rudd J.S., Kousoulas K.G., Rider P.J.F. (2021). Novel Oncolytic Herpes Simplex Virus 1 VC2 Promotes Long-Lasting, Systemic Anti-melanoma Tumor Immune Responses and Increased Survival in an Immunocompetent B16F10-Derived Mouse Melanoma Model. J. Virol..

[82] Taneja S., MacGregor J., Markus S., Ha S., Mohr I. (2001). Enhanced antitumor efficacy of a herpes simplex virus mutant isolated by genetic selection in cancer cells. Proc. Natl. Acad. Sci. USA.

